# Editorial: Inorganic Biomaterials

**DOI:** 10.3389/fbioe.2016.00002

**Published:** 2016-01-21

**Authors:** Aldo R. Boccaccini, Wolfram Höland

**Affiliations:** ^1^University of Erlangen-Nuremberg, Erlangen, Germany; ^2^Ivoclar Vivadent AG, Schaan, Liechtenstein

**Keywords:** editorial, biomaterials, bone engineering, bioactive glasses, bioceramics, dental restoration, scaffolds, coatings

**The Editorial on the Research Topic**

**Inorganic Biomaterials**

The objective of this research topic “Inorganic Biomaterials” within the scope of the article series “Bioengineering and Biotechnology” featured by the open access journal “Frontiers” was to present a comprehensive introduction to the field of inorganic bioactive biomaterials being considered for the replacement of hard tissues, in particular, bone tissue and related topics in bone tissue engineering and dental restoration.

Two important basic articles included in this research topic are written in the form of reviews. One of these articles, a review written by the inventor of Bioglass^®^ and pioneer of the field of inorganic biomaterials, Prof. Larry Hench (USA), in collaboration with Prof. J. R. Jones (UK), provides a comprehensive overview of the development and applications of the biomaterial Bioglass^®^, including a discussion on the remaining challenges for further research in the field in order to tackle current clinical needs. The second review paper included in this volume is written by one of the pioneers of the field of inorganic bioactive materials, Prof. T. Kokubo (Japan), and it covers scientific approaches to converting metal surfaces into bioactive surfaces through the formation of novel ceramic surface layers.

The subsequent publications have been coauthored by young researchers from the world’s leading research groups headed by renowned scientists in the field, including Prof. T. Kasuga (Japan), Prof. S. Best (UK), Prof. E. Verné (Italy), Prof. D. Brauer (Germany), Prof. R. Hill (UK), and Prof. A. R. Boccaccini (Germany). The aforementioned authors have focused on publishing the latest research results, and their papers cover a series of relevant topics, which include

– three-dimensional preparation and characterization of scaffolds for bone tissue engineering,
– additives to biomaterials, such as metallic ions, which have not yet been investigated fully and might significantly improve the functionality of biomaterials, e.g., cements,
– bioactive glass–polymer composite coatings,
– controlled ion release as a special type of drug delivery,
– special processes for the fabrication of composites for bone regeneration, and
– effect of porosity on bioceramic properties.

In addition to bioactive materials, inorganic biomaterials for restorative dentistry are presented. A special focus has been placed on demonstrating how several crystal phases can be precipitated in a glass–ceramic in a controlled manner. This process is called “twofold nucleation and crystallization in glasses to develop biomaterials” and the resultant biomaterials can be imparted with radiopaque characteristics, which is highly relevant when they are used for dental applications.

The editors would like to extend their gratitude to the Frontiers team in Lausanne, Switzerland, for their outstanding commitment and dedication. It has been a pleasure to create this special edition, even though it entailed intensive and concentrated work. We also thank the International Commission on Glass (ICG) which has contributed financially, via a grant to the Technical Committee 04 (Bioglasses, head: Prof. J. R. Jones), to support this publication. It is our wish that this volume will contribute to expand the knowledge in the field of inorganic biomaterials, and it will be useful not only to established researchers based both in industry and academia but also to the increasing number of young researchers starting their careers in the field.

At the very moment of writing this editorial, the sad news of the death of the inventor of Bioglass^®^ and pioneer of biomaterials research, Prof. Larry Hench, reached us, giving thus a very special timeliness character to this Frontiers topic. Both editors knew Larry personally and collaborated with him in numerous capacities during many years. Wolfram Höland would like to highlight the many scientific discussions with Prof. Hench, and numerous joint activities involving writing chapters in basic scientific books and creating a joint publication in the field of biomaterials. Aldo R. Boccaccini was a colleague of Prof. Hench for several years at Imperial College London. Through inspiring discussions and scientific exchanges, Prof. Hench became a decisive influence in Aldo R. Boccaccini’s academic career. Since his retirement from Imperial College London, Prof. Hench continued working tirelessly giving lectures, publishing research papers and books, attending conferences, and receiving a number of prizes honoring his achievements. Larry Hench was not only a brilliant materials scientist but also a wonderful and enthusiastic person with a winning personality who has inspired generations of young researchers to follow in his footsteps. His ardor to propose Bioglass^®^ for various applications in hard and soft tissue engineering will influence biomaterials research for years to come.

We dedicate this Frontiers research topic to the memory of Prof. Larry Hench.

## Author Contributions

The editorial was written jointly by the two editors of the topic.

## Conflict of Interest Statement

The authors declare that the research was conducted in the absence of any commercial or financial relationships that could be construed as a potential conflict of interest.

